# Ranking the quality of protein structure models using sidechain based network properties

**DOI:** 10.12688/f1000research.3-17.v1

**Published:** 2014-01-21

**Authors:** Soma Ghosh, Saraswathi Vishveshwara

**Affiliations:** 1 Molecular Biophysics Unit, Indian Institute of Science, Bangalore, 560012, India; 2 I.I.Sc. Mathematics Initiative, Indian Institute of Science, Bangalore, 560012, India

## Abstract

Determining the correct structure of a protein given its sequence still remains an arduous task with many researchers working towards this goal. Most structure prediction methodologies result in the generation of a large number of probable candidates with the final challenge being to select the best amongst these. In this work, we have used Protein Structure Networks of native and modeled proteins in combination with Support Vector Machines to estimate the quality of a protein structure model and finally to provide ranks for these models. Model ranking is performed using regression analysis and helps in model selection from a group of many similar and good quality structures. Our results show that structures with a rank greater than 16 exhibit native protein-like properties while those below 10 are non-native like. The tool is also made available as a web-server

( http://vishgraph.mbu.iisc.ernet.in/GraProStr/native_non_native_ranking.html), where, 5 modelled structures can be evaluated at a given time.

## Introduction

Proteins are known to take up unique well defined structures that allow them to function efficiently under a given condition ^1^. This becomes much more fascinating when one considers the time taken by a protein to fold *in vivo* ^2^. Studies over the past decades have facilitated the preparation of a blueprint of the rules that govern protein folding ^3– 6^. The roles of hydrophobic residues in structural packing, e.g. proline and glycine as helix breakers, are now very well established ^7, 8^. Details of the various pair-wise interactions that hold the structure intact are also available in the literature ^9^. However, even with the wealth of resources available, determining the structure of a protein from its amino-acid sequence still remains a challenging task.

To begin with, protein structure prediction requires understanding of the differences that exist between a well-folded protein structure and a modelled structure. Many large scale decoy structures that mimic a native protein structure, but with minor variations (such as the sidechain orientations, hydrogen bonds and so on), are now freely available ^10– 12^. Such datasets are generated using various computational approaches such as molecular dynamics ^13– 15^ and discrete state models ^16^. Decoy structures can be compared with a large number of available native structures, hence, forming an important resource to understand patterns that are unique to natively folded proteins.

For many years now, proteins structures have been represented as networks, with residues forming nodes with edges representing various factors that are important for protein structures, such as hydrogen bonds ^17^, and Cα distances ^18^. Although these networks help in understanding the structure of a protein at the level of secondary structures and backbone atoms, determining the subtle changes that occur at the level of sidechain interactions are not captured. We have been working on Protein Sidechain Network (PSN) for a number of years ^19, 20^ and have done various rigorous analyses at different levels to show its usefulness ^21– 26^. Generating networks at the level of a sidechain not only takes care of the geometry but also the chemistry that is encoded in the sidechain atoms of every amino acid in the polypeptide chain.

Support vector machine (SVM) is a machine learning algorithm mainly used for the purpose of classification ^27^. The algorithm uses a training dataset to learn patterns and finally use those patterns to classify new cases. Given the complexity of biological systems, machine learning algorithms are widely used in biology to predict cellular locations ^28, 29^, cancer tissue classifications based on gene expression data ^30– 32^ and further in cases of protein structures to identify SCOP classes ^33^, binding sites ^34, 35^ and also the quality of protein structures using features, such as secondary structures and hydrophobicity ^36, 37^.

Recently, we have demonstrated the capabilities of PSNs to distinguish native structures from decoy models. We started with comparing the network properties of PSNs from native and decoy models where we established the unique network features exhibited by native structures ^38^. This work was further followed by an in-depth analysis, where PSNs at different interaction strengths (I _min_ = 0%–7%) and SVM were used in tandem to classify the protein as native or non-native like. Further, the method was validated using a large number of CASP 10 [10 ^th^ community wide experiment on the Critical Assessment of Techniques for Protein Structure Prediction] predicted models. Overall, an accuracy of 94% was achieved by this method ^39^.

As an extension of our previous work, where a simple binary classification was carried out ^39^, here we have developed a method to rank the quality of model structures through probability estimates. This advance is particularly important in cases where one needs to select the best quality structures from a set of many similar and good quality models. Many tools have now been developed that can successfully generate many possible structure candidates from a sequence; however, predicting the best from this list is still a demanding task and needs attention. In the present study we have observed that the structures with a rank greater than 16 generally show native like properties and hence this method provides a good measure for the rank and quality of a model.

## Methods

The main aim of this work was to obtain a ranking for a set of modeled structures and to select the best modeled structure that closely resembles a native structure. To achieve this goal, we obtained a large number of native and non-native structures and generated PSNs. The network parameters from the PSNs are combined with SVM to build a mathematical model and the ranking of each structure is determined using logistic regression analysis. Details of each step are provided below.

### Datasets

Two sets of data were used for this study;

a) a positive dataset (PSN-QA_positive), that consisted of 5422 protein crystal structures with resolution < 3A, R-factor < 0.25 and PDB size > 100 This dataset was curated using PISCES ^40^,

b) a negative dataset (PSN-QA_negative) that considered different decoys as well as modelled structures from various publicly available resources and databases.

Details of the individual datasets are provided in Table 1. Finally, a total of 29543 non-native structures were obtained.

**Table 1.** List of resources from which decoy/modelled structures have been obtained.

Dataset	# decoy/modelled structures	Website
CASP3	971	http://predictioncenter.org/download_area/CASP3/
CASP7	10	http://predictioncenter.org/download_area/CASP7/
CASP8	10299	http://predictioncenter.org/download_area/CASP8/
CASP9	7711	http://predictioncenter.org/download_area/CASP9/
CASP10b	1428	http://predictioncenter.org/download_area/CASP10/
Rosetta protein decoy set	2660	http://depts.washington.edu/bakerpg/decoys/
Standard and complete collection of decoy set	1799	http://babylone.ulb.ac.be/decoys
Single decoy set	17	http://dd.compbio.washington.edu/download.shtml
Haemoglobin structural set	609	http://dd.compbio.washington.edu/download.shtml
Immunoglobulin structural set	3659	http://dd.compbio.washington.edu/download.shtml
Immunoglobulin structural hire set	380	http://dd.compbio.washington.edu/download.shtml

Table modified from ^39^

**Figure 1.**
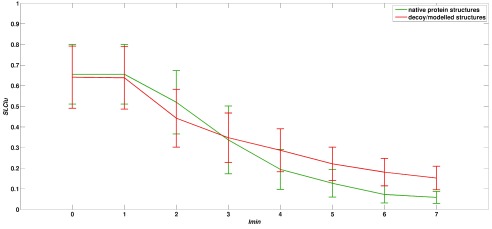
Transition profile of native protein structures and their corresponding decoy/modelled structures. Transition profile of one of the network features (SLClu; see Table 2) as a function of I _min_ is shown for 7 randomly selected native structures [green] and their corresponding decoy structures [red]. A clear distinction between the two transition profiles is visible, highlighting the 3 characteristic features that are uniquely displayed by native protein structures. X axis represents I _min_ from 0% to 7% and Y axis represents the average value of the SLClu obtained by native and decoy/modelled structures.

**Figure 2.**
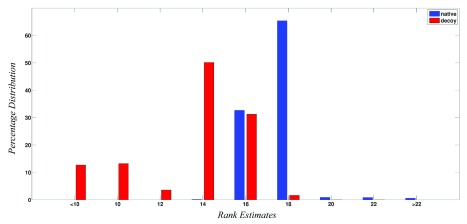
Percentage distribution of rank for native and non-native structures. The figure shows the percentage distribution of ranks for the 5422 native structures (blue) and 29543 decoy/modelled structures (red). X-axis represents ranks while Y-axis represents the percentage distribution. It is clear that native structures have higher ranks (> 16) as compared to the decoy/modelled structures.

Native and non-native structures used to generate Protein Structure Networks Dataset 1: List of 5422 native protein structures The positive dataset (PSN-QA_positive) used in this study consisted of 5422 native protein structures, that were downloaded using PISCES. Three letter codes of the PDBs are provided.
Dataset 2: Details of the decoy dataset. The negative dataset (PSN-QA_negative) used in this study were obtained from various publicly available resources.
Dataset 3: Data used to generate Figure 1 in the associated article. This file contains the value of SLClu (size of the largest cluster) for example protein structures at different Imin values (interaction strength cutoffs).
Dataset 4: Data used to generate Figure 2 in the associated article. The file contains the rank values obtained by the native and the decoy datasets. Please note that we have not kept a track of the correspondence between the Id and PDB Ids.

### Construction of the Protein Structure Network

As mentioned above, our laboratory has been working extensively on protein structure networks ^19^, specifically generated at the level of non-covalent interactions of sidechains. Details to generate PSNs are available in our previous work ^20^ and a brief description is provided here.

PSNs are generated by considering amino acids as nodes and edges are constructed between these nodes based on the non-covalent interaction strengths between them. Interaction strengths between any two residues is calculated as follows,

$$I_{ij}=\frac{n_{ij}\times 100}{\sqrt{N_{i}\times N_{j}}}\;\;\;\;\;(1)$$

where, I _ij_ = strength of interaction between residues i and j, where |i – j| ≥ 2; n _ij_ = number of distinct interacting atom pairs between i and j within a distance cut-off of 4.5 Å (excluding the backbone atoms); N _i_ and N _j_ are the normalization values for residues i and j obtained from a statistically significant dataset of proteins, as defined in our previous work ^20^. Based on the interaction strengths between these residues, PSNs can then be generated at different interaction strength cutoffs (I _min_), with a lower cutoff generating a dense network and including even the weaker interactions, while a higher cutoff signifies a network made of very strong non-covalent interactions and hence sparse. For this study, PSNs were generated at different I _min_s ranging from 0% to 7%.

Various network parameters such as number of non-covalent interactions (NCov), size of the largest cluster (SLClu), clustering coefficient (CCoe), size of the largest k-1 and k-2 communities, are calculated for each PSNs generated. Furthermore, the differences between these parameters at consecutive I _min_s are also considered in this study. In our previous studies ^39^, we have discussed the importance of the transition profile of the various network parameters as a function of I _min_ to characterize the native structures and therefore distinguish them from the non-native ones. Along with the network parameters, main chain hydrogen bonds (MHB) ^41^ were also analysed and included in the study. Table 2 provides a detailed list of all the network parameters that have been used in this study.

**Table 2.** List of network features calculated in this study.

Parameter	Description
NCov	Number of non-covalent interactions, defined by the number of edges in a PSN
SLClu	Set of connected nodes with maximum number of residues (evaluated using DFS algorithm ^44^)
Top1-ComSk1	A clique is a subset of nodes in the network, such that all nodes are connected to all other nodes. Union of k-cliques such that k-1 nodes are shared between the cliques is termed as k-1-community ^45^. This parameter represents the size of the largest k-1-community
Top2-ComSk1	Cumulative size of the top2 largest k-1-community
Top3-ComSk1	Cumulative size of the top3 largest k-1-community
ComSk2	Union of k-cliques such that k-2 nodes are termed as k-2-community. Represents the size of the largest k-2-community
Ccoe	Avg. clustering coefficient of the network, based on the algorithm given in ^46^
CCoe-LClu	Avg. clustering coefficient of the largest cluster. This was calculated by extracting the subnetwork that forms the largest cluster
CCoe-Lcomm	Avg. clustering coefficient of the largest k-2 community
d(NCov)	Represents the transition profile of non-covalent interaction as a function of I _min_
d(SLClu)	Represents the transition of the size of the largest cluster as a function of I _min_
d(ComSk2)	Represents the transition of the size of the largest k-2 community as a function of I _min_

Table adapted from ^39^

### Support Vector Machine

As described before, SVMs are machine learning algorithms that learn patterns from a training dataset and further use that pattern to classify new datasets. In this study, we have built an SVM classifier based on the patterns that are specific for a native PSN. First, we randomly divided the datasets into a training set and a test set, so that the training set contained 3000 native structures and 3000 non-native structures. Remaining structures were set aside to form the test set. This was repeated 10 times to generate 10 random test sets and training sets. Compared to our previous study, we here went one step further and used the liblinear package of LibSVM ^42, 43^, to obtain the probability estimates (using –s8 option in the liblinear package) of each data point and thereby to obtain ranks for each of them. Furthermore, since the different network parameter values have different ranges, the values were scaled between -10 to +10 before the analysis.

## Results

### Network features of PSNs

Twelve network features (at different I _min_s) ( Table 2) and MHB are combined to get a total of 94 features that best characterize a PSN. Details about these parameters and the characteristic transition curves specific to PSNs generated from native structures are discussed in detail in our previous work ^39^. Briefly, the transition profiles ( Figure 1) as obtained by plotting the network features of native protein structures as a function of I _min_ show three specific features, a) higher value at lower I _min_, (b) lower value at higher I _min_ and finally (c) steep transition between I _min_ = 1%–4%. Figure 1 shows the transition profile of 7 randomly selected native protein structures and their corresponding 981 model structures. A clear difference between the transition profiles of a native protein structure and decoy/modelled structures is visible. These differences are observed in all the datasets used in this study and forms the basis of the method developed here.

### SVM and the liblinear package

The main aim of this work was to obtain a ranking scheme for structure quality prediction. The 94 network features were combined into SVM using the liblinear package to obtain a ranking model. Specifically, for model generation, ‘L2-regularized L2-loss ranking support vector machine’ solver and cost value (c) equal to 2 was used ^43^. As mentioned in the Methods section, 10 random training and test sets were obtained and the ranking model was generated for all the train sets. Finally, the model which showed the best pairwise accuracy of 98.2% was selected for further analysis.

### Rank estimates

Figure 2 shows the percentage distribution of the ranks obtained by the 5422 native protein structures and 29543 non-native structures. These ranks represent the quality of the structures as determined by the network parameters using the SVM trained model. From figure 2, it is now quite evident that native structures almost always score above 16, while the scores of the non-native structures range from -70 to 20 with the majority being ≤ 16. It should be pointed out here that the dataset of decoy structures is taken from databases such as CASP and Rosetta and therefore in many cases might also contain structures very close to native or almost native like, thereby leading to some structure scoring beyond 16, but always ≤ 20. From Figure 2, it can now be safely assumed that structures scoring above 16 show native like properties and scores of bad, unrefined models are generally very low.

### Web-server

**Figure 3.**
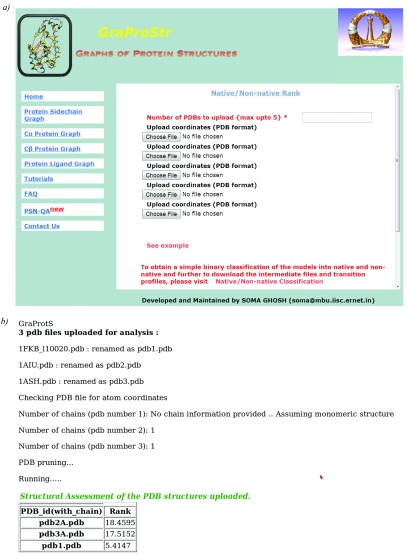
Web-server for ranking protein structures. The figure shows screenshots of the **a**) home page and **b**) results page for structure ranking. At a given time, 5 structures can be uploaded. For structures with multiple chains, each chain would be treated individually. The output would be provided in a tabular format.

**Figure 4.**
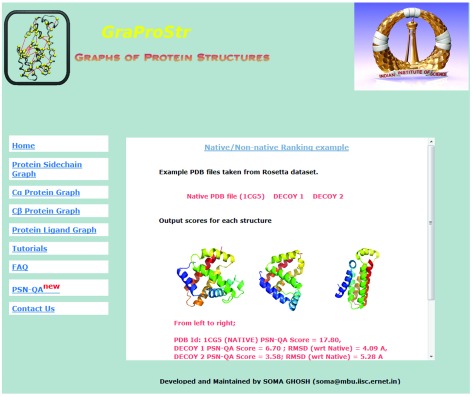
Example test case as shown in the web-server. For easy understanding, a test case of native structure (PDB Id: 1CG5) and its two decoy structures (from Rosetta) is also made available. The page shows the structures and the PSN scores obtained by them. PDB files are also available for download.

This tool is now made freely available for public use in the form of a web-server, http://vishgraph.mbu.iisc.ernet.in/GraProStr/native_non_native_ranking.html. Figure 3 shows the home page of the web-server ( Figure 3a) and the output format ( Figure 3b). A test case ( PDB Id: 1CG5 and its decoy structures from Rosetta) is also provided with its scores as an example. Figure 4 shows the screenshot of the example test case. The tool can analyse five structures at a given time. For structures with multiple chains, individual chains are treated as different structures for the analysis. The tool accepts files in PDB formats as input and outputs the ranks for each model in a tabular format.

## Discussion

Proper folding of protein structures is imparted by various energetic and topological features ^1, 3– 9^. While the secondary structures are stabilized by backbone hydrogen bonds, the mutual orientation of the secondary structures are uniquely determined by the sidechain interactions. Although studies at the backbone level have contributed enormously to the understanding of the protein structure ^17, 18^, they are not sufficient to understand the subtle balance at the atomic level. Our previous studies have highlighted the role of non-covalent interactions of the sidechain atoms in functioning ^23, 25, 26^ as well as stability ^22, 24^ of protein structures. Protein structure networks are designed to account for sidechain interactions and therefore the network captures not only the geometric but also the chemistry encoded in the sidechain.

In our earlier studies, we had exploited protein structure networks to discriminate the native structures from the non-native ones. This is mainly done at the level of sidechain with only one important feature, MHB, representing the properties of the backbone atoms. In all these studies ^38, 39^, discrimination between the two sets is done qualitatively, with the method simply classifying the structures as native or non-native. Such qualitative analysis becomes ineffective when used for closely related and almost native like structures. However, given the current state of art in the field of protein structure prediction, we believe that expertise has been attained to predict near native like structures and more work is required now to select the best structure from a set of very similar structures.

The present work is an extension of our earlier work, where we have addressed the issue described above in a quantitative manner. Here, we have built a model that would score the structures based on how closely they mimic a native structure, instead of providing a simple binary classification. We were able to use the liblinear package of libSVM to build such a model. The model was further tested on a set of 5422 native structures and 29543 decoy/modelled structures. The ranking scheme ( Figure 2) is clearly able to discriminate good structures from the bad ones. All the 5422 native structures get a rank greater than 16, while the scores for decoy/modelled structures range from -70 to 20. Overall, it can be concluded that structures with score > 16 display native like properties as evaluated from a network perspective and the models below the score of 12 are definitely show non-native like properties and do not mimic native structures.

## Conclusion

In summary, large numbers of native as well as decoy/modelled structures have been used to build an SVM model. This model was trained using 94 features that included 93 network parameters and main chain hydrogen bonds. The model has an overall accuracy of 98.2% and can successfully rank structures based on their quality as determined from protein structure networks. Generally, structures with rank > 16 display native like properties and can be regarded as good quality structures. This is an important advancement from the previous qualitative assessments and would be helpful in cases where one needs to extract the best structure from a set of closely related structures.

## Data and software availability

**Data**

Figshare: Protein Structure Network : Quality Assessment (PSN-QA), doi: 10.6084/m9.figshare.902838 ^47^.

**Software**

Protein Structure Network Quality Assessment (PSN-QA) tool: http://vishgraph.mbu.iisc.ernet.in/GraProStr/native_non_native_ranking.html
