# Supplementary material for: Ranking the quality of protein structure models using sidechain based network properties
Source: F1000Res. 2014 Jan 21;3:17. [Version 1] doi: 10.12688/f1000research.3-17.v1 (PMC4038323; doi:10.12688/f1000research.3-17.v1)
Supplement: Peer review history

# Peer review: Referee response for version 1

**Published:** 2014-05-06

**Authors:** Soumen Roy, Rajdeep Kaur Grewal

**Affiliations:** 1 Bose Institute, Kolkata, India

This paper aims at ranking protein structures in order to differentiate native protein structures from non-native/decoy models. For this, the authors employ machine learning approaches (Support Vector Machines) and assign ranks, based on regression analysis, to these models using Protein Structure Networks. This study includes the side chain interactions of amino acid residues unlike the previous network based approaches for detecting the most native-like structures from a huge set of decoys.

The authors have built upon their earlier work employing Protein Structure Networks (PSN) to differentiate the native conformations from decoy/modelled structures; PSN parameters (93 network features) along with main chain hydrogen bonds were used to built the SVM classifier. The web tool provides wide and simple accessibility of the aforementioned methods to the larger community. Non-specialists should find it useful.

We have the following comments:

- Use of network metrics – mostly based on “size” of higher communities and largest cluster, and the average clustering coefficients have been discussed by the authors. A discussion as to why these particular metrics have been chosen over so many other available network metrics would certainly be helpful for researchers with a keen interest in network theory.
- The methodology of ranking structures could certainly be presented in more detail. The part preceding *“Finally, the model which showed the best pairwise accuracy of 98.2% was selected for further analysis”* would do much better with a more detailed explanation, especially about how LibSVM is useful here.

 

It would be good to know the computational complexity as well as advantages of the present approach over other accessible packages. A table summarizing this would be a highly desirable addition especially because the authors state in the Conclusion that this is an important advancement from the previous qualitative assessments and would be helpful in cases where one needs to extract the best structure from a set of closely related structures.

We have read this submission. We believe that we have an appropriate level of expertise to confirm that it is of an acceptable scientific standard.

# Peer review: Referee response for version 1

**Published:** 2014-04-14

**Authors:** Rahul Banerjee

**Affiliations:** 1 Crystallography and Molecular Biology Division, Saha Institute of Nuclear Physics, Kolkata, India

- The title is appropriate, and the abstract represents a suitable summary of the work.
- Although the design and methodology of the calculation are appropriate for the subject under   study, some aspect of the calculation could have been explained in greater detail. This is discussed in greater detail in the report below.
- The conclusions are justified on the basis of the results obtained in the study.
- Enough information has been provided to replicate the calculations.

The authors provide a novel method to validate protein structures based on the network properties of non-bonded side chain contacts within proteins. The method could find extensive application in the structural validation of both experimentally determined protein structures by x-ray crystallography, or modeled structures. Thus as a validation tool it could prove to be an extremely valuable addition to other existing methods. The authors have also installed a web server, thus making the facility available to a wide cross section of potential users.

The success of any validation method depends on the scoring (or ranking) scheme adopted to sort structures based on some criteria. Unfortunately, the details regarding the ranking scheme are extremely terse or assume that the reader will be conversant with the details of support vector machines (SVM) and the relevant software (LibSVM). That need not be the case, as potentially work such as this should have a wide appeal. Although the authors do cite previous work, they could discuss this in somewhat greater detail. What do the terms or options used in the sentence ‘ *Specifically, for model generation, ‘L2-regularized L2-loss ranking* *support vector machine’ solver and cost value (c) equal to 2 was* *used*.’, actually signify? Why was the specific option (s8) chosen?

The authors could also compare their methodology with currently available validation packages such as Procheck or Molprobity on a small database consisting of native protein and decoys. Given the fact that experimentally determined erroneous structures occasionally seep through the currently available validation filters, this method could provide crucial information in error detection, where other methods consistently fail.

I have read this submission. I believe that I have an appropriate level of expertise to confirm that it is of an acceptable scientific standard.
